# Molecular alterations associated with improved outcome in patients with glioblastoma treated with Tumor-Treating Fields

**DOI:** 10.1093/noajnl/vdac096

**Published:** 2022-06-21

**Authors:** Manjari Pandey, Joanne Xiu, Sandeep Mittal, Jia Zeng, Michelle Saul, Santosh Kesari, Amir Azadi, Herbert Newton, Karina Deniz, Katherine Ladner, Ashley Sumrall, W Michael Korn, Emil Lou

**Affiliations:** West Cancer Center and Research Institute, Memphis, Tennessee, USA; Caris Life Sciences, Phoenix, Arizona, USA; Virginia Tech Carilion School of Medicine, Roanoke, Virginia, USA; Caris Life Sciences, Phoenix, Arizona, USA; Caris Life Sciences, Phoenix, Arizona, USA; Pacific Neuroscience Institute, Saint John’s Cancer Institute, Santa Monica, California, USA; Arizona Oncology Biltmore, Phoenix, Arizona, USA; Neuro-Oncology Center, Advent Health Cancer Institute, Orlando, Florida, USA; Division of Hematology, Oncology and Transplantation, Masonic Cancer Center, University of Minnesota, Minneapolis, Minnesota, USA; Division of Hematology, Oncology and Transplantation, Masonic Cancer Center, University of Minnesota, Minneapolis, Minnesota, USA; Levine Cancer Institute, Charlotte, North Carolina, USA; West Cancer Center and Research Institute, Memphis, Tennessee, USA; Division of Hematology, Oncology and Transplantation, Masonic Cancer Center, University of Minnesota, Minneapolis, Minnesota, USA

**Keywords:** biomarkers, genomic profiling, glioblastoma, gliomas, Tumor-Treating Fields

## Abstract

**Background:**

The genomic and overall biologic landscape of glioblastoma (GB) has become clearer over the past 2 decades, as predictive and prognostic biomarkers of both de novo and transformed forms of GB have been identified. The oral chemotherapeutic agent temozolomide (TMZ) has been integral to standard-of-care treatment for nearly 2 decades. More recently, the use of non-pharmacologic interventions, such as application of alternating electric fields, called Tumor-Treating Fields (TTFields), has emerged as a complementary treatment option that increases overall survival (OS) in patients with newly diagnosed GB. The genomic factors associated with improved or lack of response to TTFields are unknown.

**Methods:**

We performed comprehensive genomic analysis of GB tumors resected from 55 patients who went on to receive treatment using TTFields, and compared results to 57 patients who received standard treatment without TTFields.

**Results:**

We found that molecular driver alterations in NF1, and wild-type PIK3CA and epidermal growth factor receptor (EGFR), were associated with increased benefit from TTFields as measured by progression-free survival (PFS) and OS. There were no differences when stratified by TP53 status. When NF1, PIK3CA, and EGFR status were combined as a Molecular Survival Score, the combination of the 3 factors significantly correlated with improved OS and PFS in TTFields-treated patients compared to patients not treated with TTFields.

**Conclusions:**

These results shed light on potential driver and passenger mutations in GB that can be validated as predictive biomarkers of response to TTFields treatment, and provide an objective and testable genomic-based approach to assessing response.

**Key Points**

- Alterations in NF1 were associated with increased benefit from TTFields.
- Wild-type PIK3CA and EGFR also aligned with increased benefit from this approach.
- These results provide insight into molecular differences that can be validated to tailor treatment.

**Importance of the Study**

The application of Tumor-Treating Fields (TTFields) is a part of the standard-of-care approach to treating patients with glioblastoma (GB). To date, the genomic factors associated with improved or lack of response to TTFields have not been identified. In this study, we provide the first identification of a molecular signature associated with increased benefit from TTFields. This signature opens the door to a personalized treatment approach for patients with GB. The value of this study is that it provides insight into the role of comprehensive genomic profiling in uncovering potential predictive and prognostic biomarkers associated with response to TTFields.

Glioblastoma (GB) is the most common primary malignant brain tumor, accounting for approximately 50% of brain cancers.^1^ Most patients die within 1-2 years of diagnosis with a median progression-free survival (PFS) from diagnosis of 6.2-7.5 months and median overall survival (OS) of 14.6-16.7 months.^2^ Estimated 2- and 5-year survival rates of 18.5% and 6.8%, respectively.^1^ For over a decade, the standard strategy for combination therapy consisted of maximal safe surgical resection, followed by concurrent radiotherapy with daily temozolomide (TMZ) chemotherapy, followed by maintenance treatment with TMZ for 6-12 months.^3^

Over the past decade, Tumor-Treating Fields (TTFields) have emerged as a complementary treatment strategy for newly diagnosed and recurrent cases of GB following trials that demonstrated clinical activity. In the first-line setting specifically, the use of TTFields was associated with significantly higher OS compared to standard-of-care combination therapy alone.^4^ TTFields comprise a form of low-intensity alternating electric fields with intermediate frequency that interfere with and prolong cell division, resulting in apoptosis. Optimal electrical frequency for the most effective cell kill varies by tumor type. For GB, an intensity of 1-3 V/cm and frequency of 200 kHz were investigated and ultimately established as the standard set of parameters for use in patients with this tumor type. Analysis of the EF-14 trial showed that TTFields + TMZ was associated with improved PFS and OS in all subgroups regardless of age, sex, Karnofsky Performance Status (KPS), methylation status of *O*^6^-methylguanine-DNA methyltransferase (MGMT), geographic region, or extent of upfront surgical resection of the tumor.^4^

Some preclinical studies have suggested that TTFields exposure induces apoptosis by both p53-dependent and -independent mechanisms.^5,6^ What remains unknown is what genomic factors within tumors affect response to TTFields at the molecular as well as the cellular levels. To date, there is no validated predictive biomarker of response to TTFields therapy other than compliance. Testable biological markers that are predictive of efficacy of TTFields in vivo have not yet been identified. The emergence of genomic markers predictive of treatment response to cancer-directed treatments—whether they take the form of cytotoxic chemotherapies, biologic targeted agents, or immunotherapeutic agents—has formed a new landscape for the field of precision oncology. Methylation of the MGMT promoter is a well-established predictor of response to TMZ and thus prognosis of patients with GB. However, despite knowledge of this and several other driving mutations in primary central nervous system malignancies, published data on genomic correlation to response to TTFields in GB have been limited. For this study, we postulated that next-generation sequencing (NGS) of treatment-naïve GB would produce a molecular signature indicating TTFields efficacy in the first-line setting, prior to first progression. Thus, we attempted to identify whether there is a molecular subset of GB with differential response to TTFields treatment. Here, we performed retrospective evaluation of a large set of resected GB tumor samples and performed deep sequencing to uncover differences in molecular alterations in patients whose treatment had incorporated TTFields in the first-line setting.

## Methods

### Demographics and Clinical Data Collection

This study was a retrospective, multi-institutional evaluation of patients with GB treated with TTFields in the first-line setting. Data were collated from genomic profiles following biopsy or surgical resection of GB tumor specimens from 6 institutions (Barrow Neurological Institute, Arizona; Levine Cancer Institute, Charlotte, North Carolina; West Cancer Center, Memphis, Tennessee; John Wayne Cancer Center, San Diego, California; Karmanos Cancer Institute, Detroit, Michigan; Florida Hospital, Florida) between December 2014 and November 2017. Patients with high-grade gliomas who had undergone molecular profiling at Caris Life Sciences were identified and their medical records were reviewed at each participating site from which their clinicopathological features, the treatment, and outcome information were extracted, de-identified, and submitted for central analysis. For final analysis, we only included patients with GB, WHO grade 4, and excluded patients who had any treatment initiated prior to tumor profiling. Molecular profiling was performed by Caris Life Sciences, as described in detail below. At each participating institution, clinical records of patients who received TTFields treatment as part of their treatment plan were reviewed and pre-specified data points were recorded by study co-investigators; a control cohort of similar size as the TTFields-treated cohort was also reviewed and recorded. The manufacturer of the device, Novocure, had no role in the identification of patients, compliance, data analysis, or any other part of this research study. No funding was acquired for this study.

### Molecular Profiling

All tumor samples were tested with comprehensive molecular profiling which included NGS on DNA and RNA as well as MGMT promoter methylation testing by pyrosequencing. NGS was performed on genomic DNA isolated from formalin-fixed paraffin-embedded (FFPE) tumor samples using the NextSeq platform (Illumina, Inc., San Diego, CA). A custom-designed SureSelect XT assay was used to enrich 592 whole-gene targets (Agilent Technologies, Santa Clara, CA). All variants were detected with >99% confidence based on allele frequency and amplicon coverage, with an average sequencing depth of coverage of >500 and an analytic sensitivity of 5%. Prior to molecular testing, tumor enrichment was achieved by harvesting targeted tissue using manual microdissection techniques. Genetic variants identified were interpreted by board-certified molecular geneticists and categorized as “pathogenic,” “likely pathogenic,” “variant of unknown significance,” “likely benign,” or “benign,” according to the American College of Medical Genetics and Genomics (ACMG) standards. When assessing mutation frequencies of individual genes, “pathogenic” and “likely pathogenic” were counted as mutations while “benign”, “likely benign” variants, and “variants of unknown significance” were excluded.

For gene fusion detection, anchored multiplex PCR was performed for targeted RNA sequencing using the ArcherDx fusion assay (Archer FusionPlex Solid Tumor panel). The FFPE tumor samples were microdissected to enrich the sample to ≥20% tumor nuclei, and mRNA was isolated and reverse transcribed into complementary DNA (cDNA). Unidirectional gene-specific primers were used to enrich for target regions, followed by NGS (Illumina MiSeq platform). Targets included 52 genes, and the full list can be found at http://archerdx.com/fusionplex-assays/solid-tumor.

MGMT promoter methylation was evaluated by pyrosequencing. DNA extraction from paraffin-embedded tumor samples was performed for subsequent pyrosequencer-based analysis of 5 CpG sites (CpGs 74-78). All DNA samples underwent a bisulfite treatment and were PCR amplified with primers specific for exon 1 of MGMT (GRCh37/hgl9 − chr10: 131 265 448-131 265 560). Methylation status of PCR-amplified products is determined using the PyroMark system. Samples with ≥7% and <9% methylation are considered to be equivocal or gray zone results.

Immunohistochemistry (IHC) was performed on full FFPE sections of glass slides. Slides were stained using automated staining techniques according to the manufacturer’s instructions and were optimized and validated per CLIA/CAO and ISO requirements. The primary antibody used against PD-L1 was SP142 (Spring Biosciences). The staining was regarded as positive if its intensity on the membrane of the tumor cells was >=2+ (on a semiquantitative scale of 0-3: 0 for no staining, 1+ for weak staining, 2+ for moderate staining, or 3+ for strong staining) and the percentage of positively stained cells was >5%. The antibody for PD-1 was NAT105 (Cell Marque) and >=1 TIL/HPF was considered positive.

### Statistical Analysis

Patient PFS was calculated from the date of patients’ glioma histological diagnosis to the first progression after TTFields treatment start in the experimental arm; and to the first progression after first-line treatment start in the control arm. OS was calculated from the patients’ histological diagnosis till patient death or last date of contact. Kaplan-Meier estimates of the PFS and OS were performed on censored data using Cox proportional hazards (PH) model. Hazard ratio and *P* values were calculated for inter-group comparisons and *P* < .05 was considered significant. Biomarker and clinicopathological features in the TTFields and control group were compared using Fisher’s exact test.

### Ethics

This study was conducted in accordance with guidelines of the Declaration of Helsinki, Belmont report, Good Clinical Practice, REMARK, and U.S. Common Rule. In keeping compliance with policy 45 CFR 46.101(b)(4), the part of this study utilizing the Caris dataset was performed using retrospective, de-identified clinical data. Therefore, this part was considered Institutional Review Board (IRB) exempt and no patient consent was necessary.

## Results

### Patient Characteristics and Survival

Data were collected from a total of 148 patients treated at 6 participating institutions; patients with grade III tumor at diagnosis, or with GB treated in the recurrent setting (a total of 36 patients) were excluded. Fifty-five patients treated with TTFields, and 57 treated with standard-of-care treatment without TTFields, were included for final analysis. Demographic characteristics were well balanced in the 2 cohorts (Table 1). Treatment regimens for both the TTFields-treated and control group patients were dominated by the use of standard-of-care concurrent chemoradiation using daily TMZ chemotherapy, followed by 5-day TMZ used in 28-day cycles for 6-12 months; some patients also or instead received different chemotherapeutic or biologic agents on or off clinical trials (Table 2). All patients not receiving TTFields were included in the control cohort for the purpose of this analysis. In patients treated with TTFields, the average duration of use of TTFields was 198 days (IQR: 52-149 days); average compliance of use of the device was 57%, with median use of 60%.

**Table 1.** Baseline Patient Characteristics

	TTFields-treated Patients, N (%)	Control Patients, N (%)
Patient, N	55	57
Gender		
Female	17 (31%)	23 (40%)
Male	38 (69%)	34 (60%)
Age		
Median age	59	58
Age range	26-79	17-75
Histology	All glioblastoma	All glioblastoma
Tumor grade	All IV	All IV
Primary tumor location		
Temporal lobe	16 (29%)	10 (18%)
Frontal lobe	14 (25%)	17 (30%)
Parietal lobe	9 (16%)	12 (21%)
Brain, NOS	12 (22%)	17 (30%)
Cerebellum	1 (2%)	0
Occipital lobe	2 (4%)	0
Thalamus	1 (2%)	1 (2%)
IDH1/2 mutation % (N)	5 (9%)	3 (5%)
MGMT methylation % (N)	24/53 (45%)	26/56 (46%)

Abbreviations: IDH, isocitrate dehydrogenase; MGMT, *O*^6^-methylguanine-DNA methyltransferase; NOS, not otherwise specified; TTFields, Tumor-Treating Fields

**Table 2.** Treatments Administered for the TTFields and Control Groups

TTFields-treated arm (n = 55)	Treatment administered prior to TTFields therapy	
	Radiation/temozolomide combination	54
	Bevacizumab	5
	Vitamin C	2
	CPT-11	2
	Nivolumab	1
	Concurrent treatment with TTFields	
	Temozolomide	41
	Bevacizumab	13
	Carboplatin	4
	Nivolumab	2
	CPT-11	1
	None	3
	Duration of Optune use	
	Average	198 days
	IQR	52-249 days
	Compliance %	57% (11%-95%)
Control arm (n = 57)	Temozolomide	56
	Bevacizumab	23
	Carboplatin	17
	Nivolumab/pembrolizumab	10
	CPT-11	4
	CCNU	5

Abbreviations: CCNU, CCNU, 1-(2-Chloroethyl)-3-cyclohexyl-1-nitrosourea (Lomustine); IQR, interquartile range; TTFields, Tumor-Treating Fields

**Figure 1.**
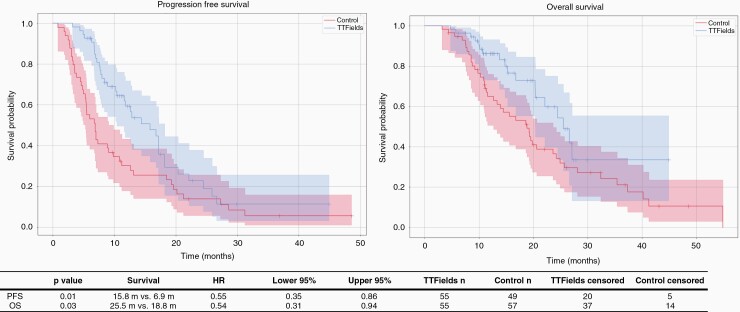
Progression-free and overall survivals of patient cohorts receiving TTFields vs control cohorts. Abbreviation: TTFields, Tumor-Treating Fields

In TTFields-treated patients, PFS was 15.8 months as compared to 6.9 months in control patients (HR = 0.55; 95% CI: 0.35-0.86; *P* = .01); OS was 25.5 months in TTFields-treated patients vs 18.8 months in the control group (HR = 0.54; 95% CI: 0.31-0.94; *P* = .03) (Figure 1). As expected, among TTFields-treated patients, those with >50% use of the device per 24-hour period had better PFS (12.6 vs 6.9 months, *P* = .0274) and also OS (not reached vs 18.8 months, *P* = .0041) than patients in the control cohort (Supplementary Figure 1). Corresponding data for TTFields-treated patients using the device at the usage rate of >75% are shown in Supplementary Figure 2.

### In Search of Genomic Biomarkers Predictive of Survival in Patients Receiving TTFields

**Figure 2.**
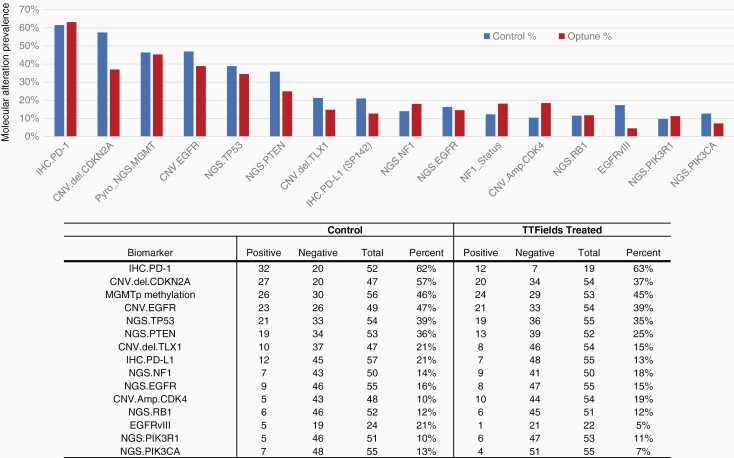
Distribution of biomarker alterations detected in GB treated with TTFields vs control treatment. Abbreviations: GB, glioblastoma; TTFields, Tumor-Treating Fields

A wide distribution of molecular alterations was detected using IHC for detection of PD-1 and PD-L1, NGS (592 genes), and pyrosequencing for detection of MGMT promoter methylation (Figure 2). The most commonly detected molecular characteristics were PD-1 expression, CDKN2A mutation, MGMT promoter methylation, epidermal growth factor receptor (EGFR) amplification, and TP53 mutation. However, for these alterations, there were no significant differences between the TTFields vs control group. MGMT promoter methylation was detected in 45% (24/53) of patients in the TTFields group and in 46% (25/56) in the control cases.

**Figure 3.**
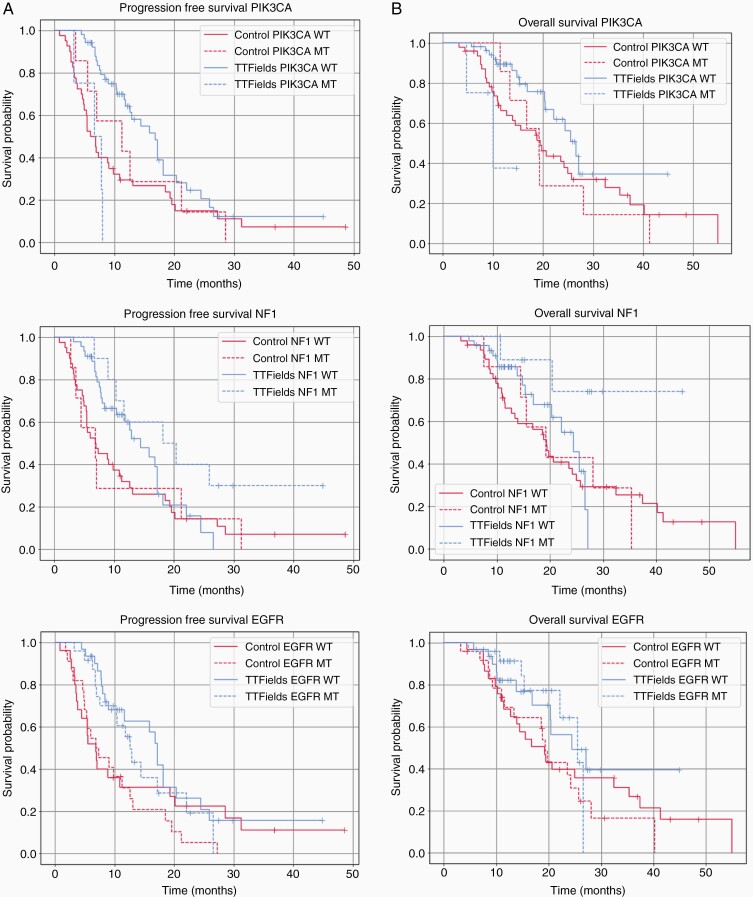
PFS (A) and OS (B) of patients treated with TTFields vs control stratified for alterations of PIK3CA (top panels), NF1 (middle panels), and EGFR alterations (lower panels). Abbreviations: EGFR, epidermal growth factor receptor; NF1, neurofibromatosis type 1; OS, overall survival; PFS, progression-free survival; TTFields, Tumor-Treating Fields

Molecular alterations were surveyed for individual association with PFS and OS in TTFields-treated and control patients. We found that TP53 mutations did not have any effect on outcome upon TTFields therapy (PFS: HR = 1.11, *P* = .7685; OS: HR = 1.14, *P* = .8000). However, alterations in PIK3CA, NF1, or EGFR, in particular, did demonstrate a trend toward different effects in TTFields-treated tumors (Figure 3; Supplementary Figure 3; Supplementary Table 1). Activating mutations in the PIK3CA gene regulate the phosphatidylinositol 3-kinase signaling cascade in many cancer types, including in infiltrative gliomas; these mutations have been associated with more disseminated versions of the disease at the time of diagnosis, as well as earlier recurrence.^7^ Specifically, our data suggested that mutation in the PIK3CA gene predicts a poor response of the tumor in general, possibly associated with decreased or lack of response to TTFields: among TTFields-treated patients, those carrying PIK3CA mutations had a significantly shorter PFS (6.7 vs 16.8 months, Cox PH *P* = .0008) and OS (10.0 vs 26.6 months; Cox PH *P* = .0158) compared to those that were wild type for PIK3CA mutation. This difference was not seen in the control group (11.2 vs 6.0 months PFS, Cox PH *P* = .6541; OS 19.2 vs 19.5 months; *P* = .6695). As is shown in Figure 3, for the first 0-20 months, there was a notable improvement in PFS for patients with wild-type PIK3CA treated with TTFields compared to control treatment; however, by 30 months, there was no significant difference in any of the 4 groups (Figure 3; Supplementary Figure 3A; Supplementary Table 1). A similar comparison was seen for OS in comparison of these groups (Figure 3; Supplementary Figure 3B; Supplementary Table 1).

Neurofibromatosis type 1 (NF1) is a tumor suppressor that encodes neurofibromin, a protein prevalent in neurons and astrocytes. Alterations in NF1 predispose to increased risk of central nervous system tumors, of which gliomas are the most common subtype in this patient population. NF1 mutations are prevalent in both de novo and transformed GB, in the form of both mutations and deletions.^8^ Here, we found that in the TTFields-treated group, NF1 alterations were associated with a response to TTFields therapy, as compared to tumors with wild-type NF1(PFS 18.2 vs 14.4 months; *P* = .07 and OS NR vs 24.7 months; *P* = .0415). In the control group, no difference was observed in patients whose tumors harbored the NF1 alterations vs wild-type NF1 (PFS 6.9 vs 6.9 months, *P* = .8933 and OS 19.3 vs 19.2 months; *P* = .8902) (Figure 3).

EGFRvIII is a truncated form of the EGFR seen in over 50% of cases of GB; other less common variants of EGFR have also been reported, including amplifications and gene fusions, in addition to the vIII form.^9^ EGFR wild-type tumors showed a trend for higher PFS with TTFields therapy 17.2 vs 12.6 months for tumors that harbored any alterations even though this finding was not statistically significant (HR = 0.517, *P* = .3628). When comparing patients with EGFR alterations receiving TTFields vs no TTFields, EGFR-intact patients had a PFS 4.6 months longer than EGFR-altered patients (*P* = .36) while the PFS was practically identical in the control patients (Figure 3; Supplementary Figure 3; Supplementary Table 1). We also evaluated TP53 alterations and found no significant differences in either PFS or OS in patients treated with TTFields in this subset. Other markers considered important in cell cycle checkpoint control, including CDKN2A, TP53, RB1, and CDK2 amplification, were not found to be associated with benefit from TTFields treatment.

Based on the above findings, we investigated the correlation and interdependency of TTFields treatment and the statuses of PIK3CA (mutations), NF1 (mutation and copy number alterations), and EGFR (EGFRvIII, fusions, and amplifications) biomarkers using Spearman’s rank correlation. The correlation coefficient between TTFields, PIK3CA, NF1, and EGFR was close to 0, suggesting that these variables were not dependent and can be used in the same model and their interactions could be examined in the model (Supplementary Figure 4). Cox PH model was used for survival regression. Linear models were built on the 4 variables, while interaction terms were applied for 2 variables (therapy and one biomarker). The models were assessed and selected based on their log-likelihood on the fitted data, and the concordance index, which is a generalization of AUC. The models with higher log-likelihood and concordance index were considered to have better predictive power and fit. Based on the model’s log-likelihood and concordance index, the one with all 4 variables had the best predictive power and fit (Supplementary Figure 5). Therefore, we further built a Molecular Survival Score (MSS) based on the combination of PIK3CA, NF1, and EGFR.

**Figure 4.**
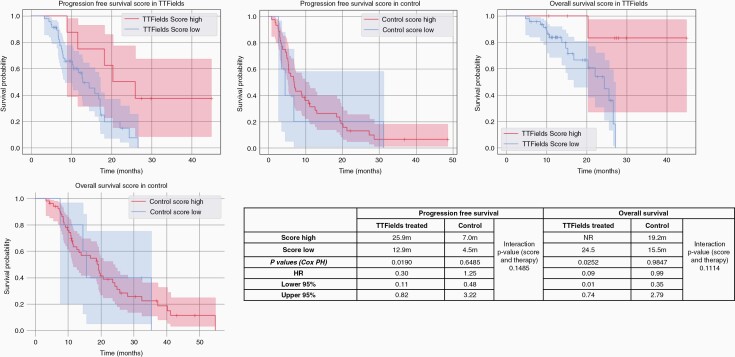
Stratification of PFS and OS based on a combined Molecular Survival Score (MSS) for comparison of PFS and OS between TTFields vs control groups. The OS, PFS, and statistical comparisons in all patients are shown here. Abbreviations: OS, overall survival; PFS, progression-free survival; TTFields, Tumor-Treating Fields

The combined MSS for each patient was calculated as follows: score of +1 was assigned for unaltered PIK3CA (wild type), EGFR (intact, wild-type with no fusion), and NF1 alteration, respectively; the reverse-scored as 0 for each factor. A sum of the scores assigned to the 3 biomarkers was considered the Survival Score of each patient, with a final range of 0-3. Score of 0-2 was considered Score Low, and score of 3 was considered Score High. Analysis of OS showed that in patients with a high MSS, the OS was not reached (95% CI: 11.6-23.6 months) for those treated with TTFields and 19.2 months (95% CI: 7.6-35.4 months) in those not treated with TTFields. For patients with Score Low, OS was 25.5 months (95% CI: 16.8-26.6 months) for those treated with TTFields and 15.6 months (95% CI: 11.6-23.6 months) for those not treated with TTFields. There is a statistically significant difference in PFS (*P* = .019) and OS (*P* = .0252) comparing MSS-high vs low patients when treated with TTFields while the effect is not seen in control arms; the interaction *P* values were trending for both PFS and OS (Figure 4). When further examining differences in OS and PFS stratified by compliance (minimum use >50% within an average 24-hour period), then the score remained predictive of PFS (*P* = .034) in TTFields-treated patients with high vs low scores in this scenario but not for OS (Supplementary Figure 6). Among TTFields-treated patients, those with tumors with MSS of 3 tend to have higher PFS and OS than patients in the control cohort.

As isocitrate dehydrogenase (IDH) mutations profoundly affect patients’ survival, we excluded IDH mutants in a subgroup analysis and confirmed all observations in IDH-WT group (Supplementary Figure 7).

## Discussion

The data presented in this study show that the presence of NF1 mutation, EGFR wild type, and PIK3CA wild type are suggestive of improved response to TTFields, as compared to patients whose tumors do not manifest this profile. Furthermore, a combination of these factors, which we designate as the TTFields MSS, provides a first attempt to create a robust tool to predict the impact of TTFields on survival in patients with GB. In this study, we uncovered initial evidence of a molecular signature associated with tumor response to therapy with TTFields.

TTFields are approved for the treatment of GB in the newly diagnosed and also in the recurrent setting. This therapeutic modality has increasingly been adopted as complementary to standard-of-care following maximal safe surgical resection and concurrent chemoradiation, and concurrent with adjuvant chemotherapy; at the same time, a number of patients go on clinical trials foregoing this treatment and there have been concerns raised about the quality of life, burden of care, and costs. These factors can be barriers in the use of TTFields therapy which has significantly improved OS when incorporated into first-line treatment algorithms following GB diagnosis. Identification of a molecular signature associated with the tumor response to this treatment is a promising decision-making tool.

For more than a decade, the presence of MGMT promoter methylation has been considered the prototype predictive biomarker of response to chemotherapy (TMZ) in GB, and it is a standard biomarker routinely tested in all patients with GB. MGMT promoter is methylated in about 35%-50% of newly diagnosed GBs^10^; in this dataset, 45% (24/53) of patients in the TTFields group and 46% (25/56) in the control had tumors that were MGMT methylated, thus consistent with wider reports in other case series. There is increased interest and understanding of the impact of genetic alteration in IDH1 and 2 genes, which in many cases is a better determinant of outcomes than histologic grades, this has led to changes in the 2016 and now the 2021 World Health Organization (WHO) classification of gliomas.^11,12^ IDH-mutated GB are typically associated with better prognosis, about 5% of GB are IDH-mutated.^13^ In our dataset, 5 patients in the TTFields arm and 3 in the control arm had IDH mutations.

Analysis of gene expression can be utilized to reveal how varying cancers may be affected by TTFields and could signify treatment responsiveness. TP53 mutation status can influence the response to certain treatments and is linked to a worse prognosis. In a GB study, 4 cell lines were used with varying TP53 status to influence TTFields treatment.^14^ Genes associated with cell cycle, cell death, and immune response were analyzed after TTFields application and were altered despite TP53 status.^14^ In our study, there was no difference detected in TTFields response between tumors that were TP53 wild-type vs mutant. TP53 is a ubiquitous tumor suppressor marker so it may not be surprising that no differences were found. However, mutant NF1, which is more glioma-associated than TP53, was associated with differential response to TTFields, and this signal pointed toward a composite score that, if validated, could help tailor therapeutic decision-making in the future.

Until the past few years, compliance in device usage over time had been the only factor associated with improved survival with TTFields. However, in the era of better access, affordability, and knowledge regarding utility of comprehensive genomic profiling in oncology, in general, including in neuro-oncology, emerging targets are being identified and are providing further insight into TTFields response mechanisms. The mechanism of action of TTFields seems to be cell cycle-dependent. Dono et al recently reported results from a retrospective analysis that detected higher post-progression survival of 14 patients with PTEN-mutated recurrent GBs who received treatment with TTFields, compared to 15 patients with PTEN wild-type tumors (22.2 vs 11.6 months, respectively). Studies like that one and the one we present here provide initial glimpses in the molecular biology of tumors that can either be affected by TTFields therapeutically, or otherwise provide a tumor microenvironment that is most susceptible to disruption via the alternating electric fields treatment strategy.

To date, most of the focus on mechanism(s) of action of TTFields has focused on basic cell biology, most notably on effects on microtubules and cell division. The next frontier of basic science research in this field is the interplay of tumor genomics, most especially of driver mutations, in laying the groundwork for a tumor microenvironment that lends itself to increased or decreased susceptibility to cell disruption at the biophysical level. In a study with non-small cell lung cancer (NSCLC) cells, there was a division of more responsive cell lines and less responsive cell lines based on the treatment of TTFields. Previous studies used gene expression analysis to ascertain molecular changes after treatment of TTFields and suggested that certain genes were altered in both the less responsive cell lines and more responsive cell lines.^15^ Ingenuity pathway analysis (IPA) was performed to determine the canonical pathways involved in the altered genes. Results suggested alterations happened in cell cycle and mitotic pathways.^15^ Manifested by the IPA results, downregulation of BRCA1 DNA damage response pathway was significant.^15^ These proteins involved in the pathway are important for double-stranded breaks within DNA, which could indicate reduced DNA repair in TTFields-treated cells.^15^ Untapped aspects and unknown factors that may merit further investigation relating to TTFields including BRCA and other damage response pathway effectors, and other genetic abnormalities including mismatch repair deficits and tumor mutation burden, which have to date been associated with response to immune checkpoint inhibitors.

Limitations of this study include potential for selection bias inherent in any retrospective study, and relatively small numbers as compared to standard prospective studies. The years that the patients examined in this study were diagnosed and treated (2014-2017) represent the beginning of the era in which TTFields began to be adopted more widely internationally in this patient population; nonetheless, more widespread acceptance of TTFields therapy in the first-line setting for GB took hold more widely following that time period. Multiple institutions were involved to maximize the number of cases analyzed; however, a number of patients from those participating institutions were alternately enrolled in clinical trials and thus did not receive treatment with TTFields at that time. The patient population we were able to examine represents those patients who were both treated with or without TTFields and also had tumors sent for genomic profiling to Caris Life Sciences. During that time period (2014-2017), comprehensive genomic profiling was not as offered as often to patients with GBs. Since that time, comprehensive genomic profiling has become more prevalent for patients with GB. In addition, as pointed out by a reviewer, patients treated with TTFields, in 2014-2017 as well as now, tend to have better performance status as compared to patients who may not receive this treatment based on physician assessment. While this study did not comprehensively evaluate additional factors associated with outcome in this disease, such as extent of resection, use of steroids at baseline or throughout the treatment course, etc., these and other factors can be taken into consideration of design of future larger-scale studies will further delve into validation of the potential biomarkers we identify here. Such studies will be likely to uncover new ones as accuracy and sensitivity of testing improves even further through whole transcriptome analysis and other methods.

In summary, this retrospective study provides indication that there are potential genomic predictors of response to TTFields treatment of patients with GB. As GB is the most common tumor type for which this technology is in widespread use at the current time, our finding that the combination of NF1 mutation, EGFR wild type, and PIK3CA wild type, formulated as a MSS, may be predictive of heightened response to TTFields warrants further investigation, including inclusion as a correlative biomarker in large-scale prospective clinical trials that will be even more robust in the era of more widespread use of TTFields and also comprehensive genomic profiling.

## Supplementary Material

vdac096_suppl_Supplementary_Material

vdac096_suppl_Supplementary_Table_S1

vdac096_suppl_Supplementary_Legends

## References

[1] Ostrom QT , Cioffi G, Gittleman H, et al. CBTRUS statistical report: primary brain and other central nervous system tumors diagnosed in the United States in 2012-2016. Neuro Oncol. 2019;21(Suppl 5):v1–v100. 31675094 10.1093/neuonc/noz150 PMC6823730

[2] Adamson C , Kanu OO, Mehta AI, et al. Glioblastoma multiforme: a review of where we have been and where we are going. Expert Opin Investig Drugs. 2009;18(8):1061–1083. 10.1517/13543780903052764 19555299

[3] Stupp R , Mason WP, van den Bent MJ, et al. Radiotherapy plus concomitant and adjuvant temozolomide for glioblastoma. N Engl J Med. 2005;352(10):987–996. 15758009 10.1056/NEJMoa043330

[4] Stupp R , Taillibert S, Kanner A, et al. Effect of tumor-treating fields plus maintenance temozolomide vs maintenance temozolomide alone on survival in patients with glioblastoma: a randomized clinical trial. JAMA. 2017;318(23):2306–2316. 29260225 10.1001/jama.2017.18718 PMC5820703

[5] Schneiderman RS , Voloshin T, Giladi M, et al ATPS-25: p53 status dependence of tumor treating fields (TTFields) efficacy against glioma cancer cells. Neuro Oncol. 2015; 17:v23.

[6] Alizadeh AA , Aranda V, Bardelli A, et al. Toward understanding and exploiting tumor heterogeneity. Nat Med. 2015;21(8):846–853. 26248267 10.1038/nm.3915 PMC4785013

[7] Tanaka S , Batchelor TT, Iafrate AJ, et al. PIK3CA activating mutations are associated with more disseminated disease at presentation and earlier recurrence in glioblastoma. Acta Neuropathol Commun. 2019; 7(1):66. 31036078 10.1186/s40478-019-0720-8 PMC6487518

[8] Lobbous M , Bernstock JD, Coffee E, et al. An update on neurofibromatosis type 1-associated gliomas. Cancers. 2020; 12(1):1–15. https://www.mdpi.com/2072-6694/12/1/114. 10.3390/cancers12010114 PMC7017116 31906320

[9] Felsberg J , Hentschel B, Kaulich K, et al. Epidermal growth factor receptor variant III (EGFRvIII) positivity in EGFR-amplified glioblastomas: prognostic role and comparison between primary and recurrent tumors. Clin Cancer Res. 2017;23(22):6846–6855. 28855349 10.1158/1078-0432.CCR-17-0890

[10] Mellai M , Monzeglio O, Piazzi A, et al. MGMT promoter hypermethylation and its associations with genetic alterations in a series of 350 brain tumors. J Neurooncol. 2012;107(3):617–631. 22287028 10.1007/s11060-011-0787-y

[11] Brat DJ , Aldape K, Colman H, et al. cIMPACT-NOW update 3: recommended diagnostic criteria for “Diffuse astrocytic glioma, IDH-wildtype, with molecular features of glioblastoma, WHO grade IV”. Acta Neuropathol. 2018;136(5):805–810. 30259105 10.1007/s00401-018-1913-0 PMC6204285

[12] Louis DN , Perry A, Wesseling P, et al. The 2021 WHO classification of tumors of the central nervous system: a summary. Neuro Oncol. 2021;23(8):1231–1251. 34185076 10.1093/neuonc/noab106 PMC8328013

[13] Yan H , Parsons DW, Jin G, et al. IDH1 and IDH2 mutations in gliomas. N Engl J Med. 2009;360(8):765–773. 19228619 10.1056/NEJMoa0808710 PMC2820383

[14] Lee YJ , Seo HW, Baek JH, Lim SH, Hwang SG, Kim EH. Gene expression profiling of glioblastoma cell lines depending on TP53 status after tumor-treating fields (TTFields) treatment. Sci Rep. 2020;10(1): 12272. 32704022 10.1038/s41598-020-68473-6 PMC7378235

[15] Karanam NK , Srinivasan K, Ding L, Sishc B, Saha D, Story MD. Tumor-treating fields elicit a conditional vulnerability to ionizing radiation via the downregulation of BRCA1 signaling and reduced DNA double-strand break repair capacity in non-small cell lung cancer cell lines. Cell Death Dis. 2017;8(3):e2711. 28358361 10.1038/cddis.2017.136 PMC5386539

