# Corrigendum to: Molecular alterations associated with improved outcome in patients with glioblastoma treated with Tumor-Treating Fields

**DOI:** 10.1093/noajnl/vdac164

**Published:** 2022-10-28

This is a corrigendum to: Manjari Pandey, Joanne Xiu, Sandeep Mittal, Jia Zeng, Michelle Saul, Santosh Kesari, Amir Azadi, Herbert Newton, Karina Deniz, Katherine Ladner, Ashley Sumrall, W Michael Korn, Emil Lou, Molecular alterations associated with improved outcome in patients with glioblastoma treated with Tumor-Treating Fields, *Neuro-Oncology Advances*, Volume 4, Issue 1, January–December 2022, vdac096, https://doi.org/10.1093/noajnl/vdac096

In the originally published version of this manuscript, PIK3CA MT vs WT, NF1 MT vs WT, and EGFR WT vs MT were incorrectly labeled in Figure 3.

PFS values for PIK3CA MT and WT were incorrectly labeled in Supplementary Table 1.

These errors have been corrected.

