# Supplementary material for: Molecular alterations associated with improved outcome in patients with glioblastoma treated with Tumor-Treating Fields
Source: Neurooncol Adv. 2022 Jun 21;4(1):vdac096. doi: 10.1093/noajnl/vdac096 (PMC9270729; doi:10.1093/noajnl/vdac096)

Supplemental Figure 1

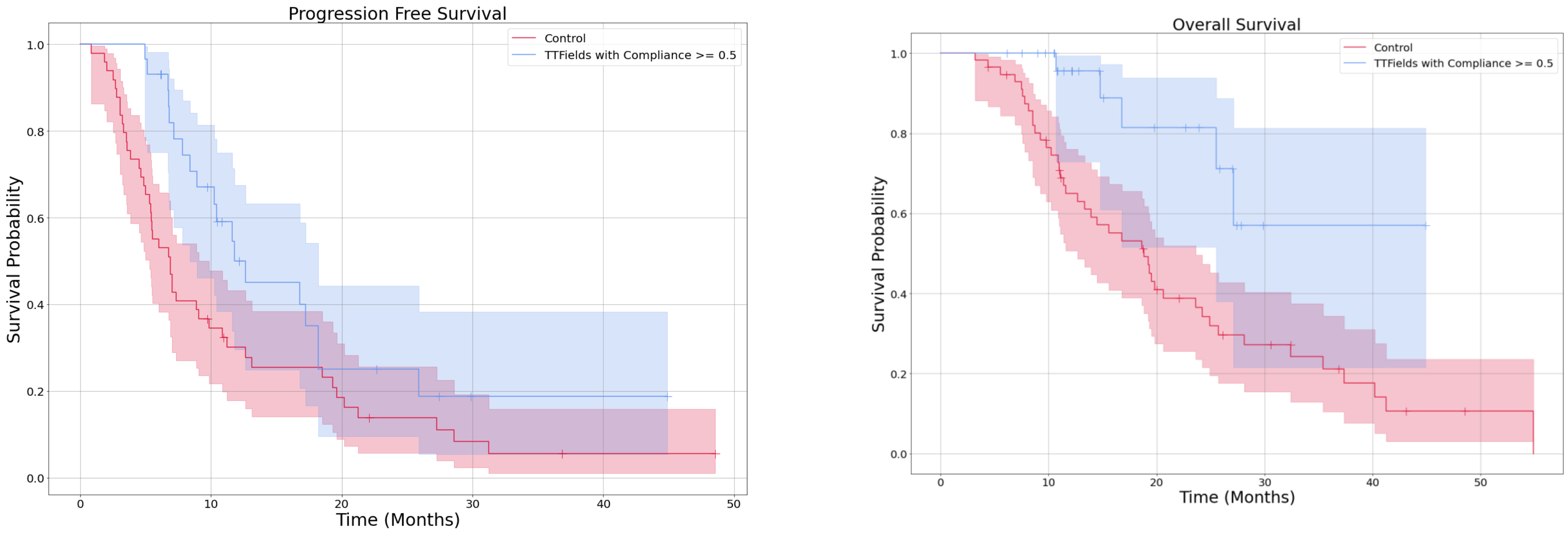

	p value	Survival	HR	Lower 95%	Upper 95%	TTFields n	Control n	TTFields censored	Control censored
PFS 0.5	0.0274	12.6m vs. 6.9m	0.54	0.32	0.93	29	49	10	5
OS 0.5	0.0041	18.8 m vs. NR	0.26	0.10	0.65	29	57	24	14

Supplemental Figure 2

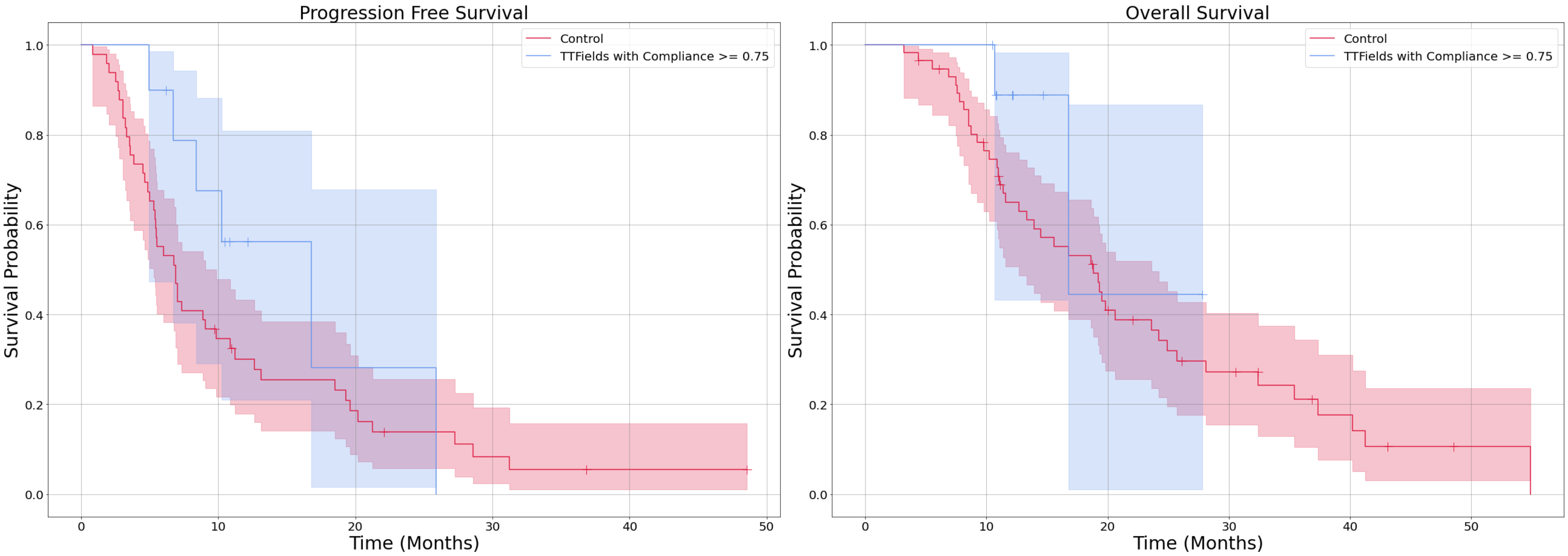

	p value	Survival	HR	Lower 95%	Upper 95%	TTFields n	Control n	TTFields censored	Control censored
PFS 0.75	0.23	16.8 m vs. 6.9m	0.59	0.25	0.39	10	49	4	5
OS 0.75	0.25	16.8m vs. 18.8m	0.43	0.10	1.81	10	57	8	14

Supplemental Figure 3A

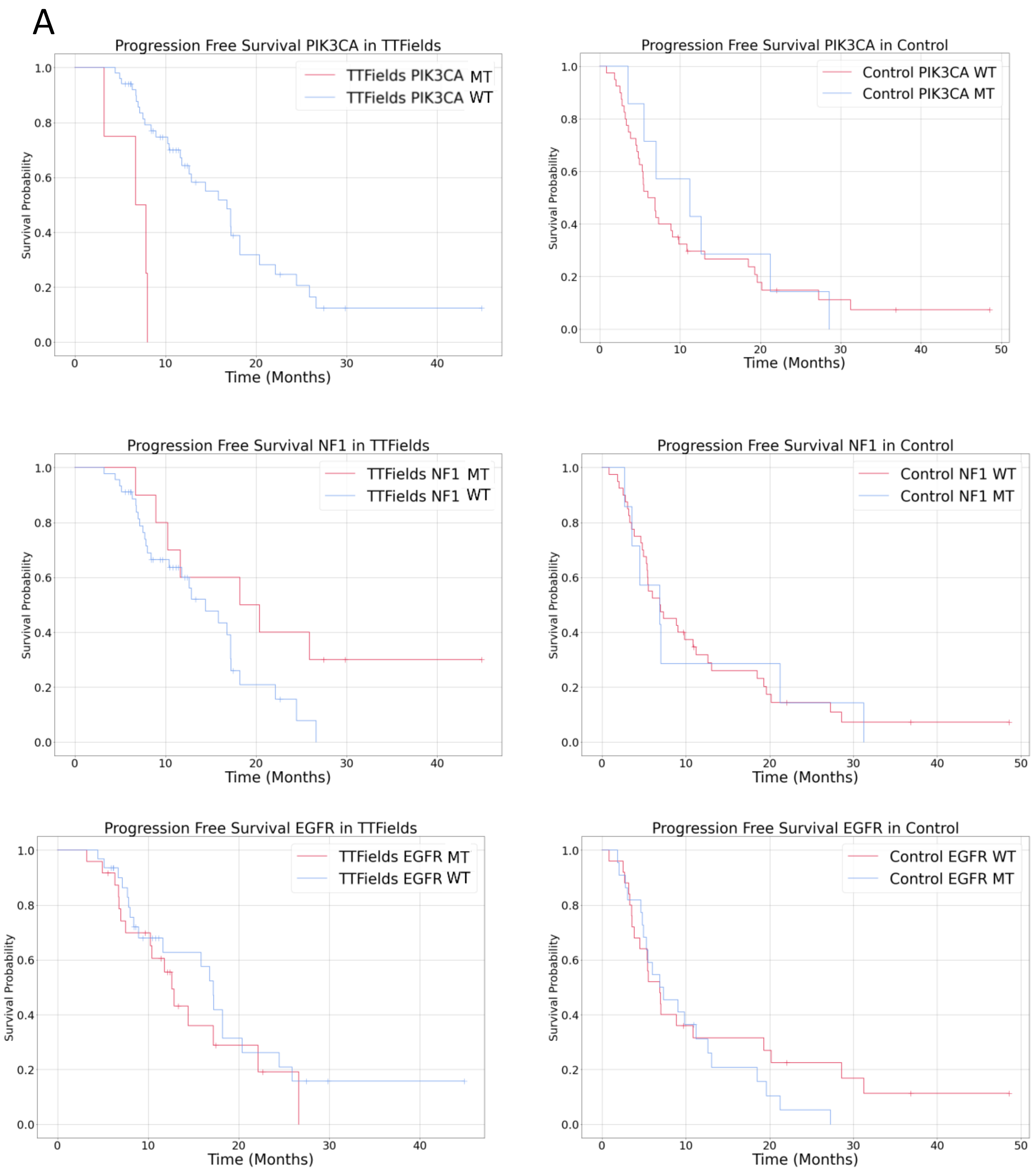

Supplemental Figure 3B

B

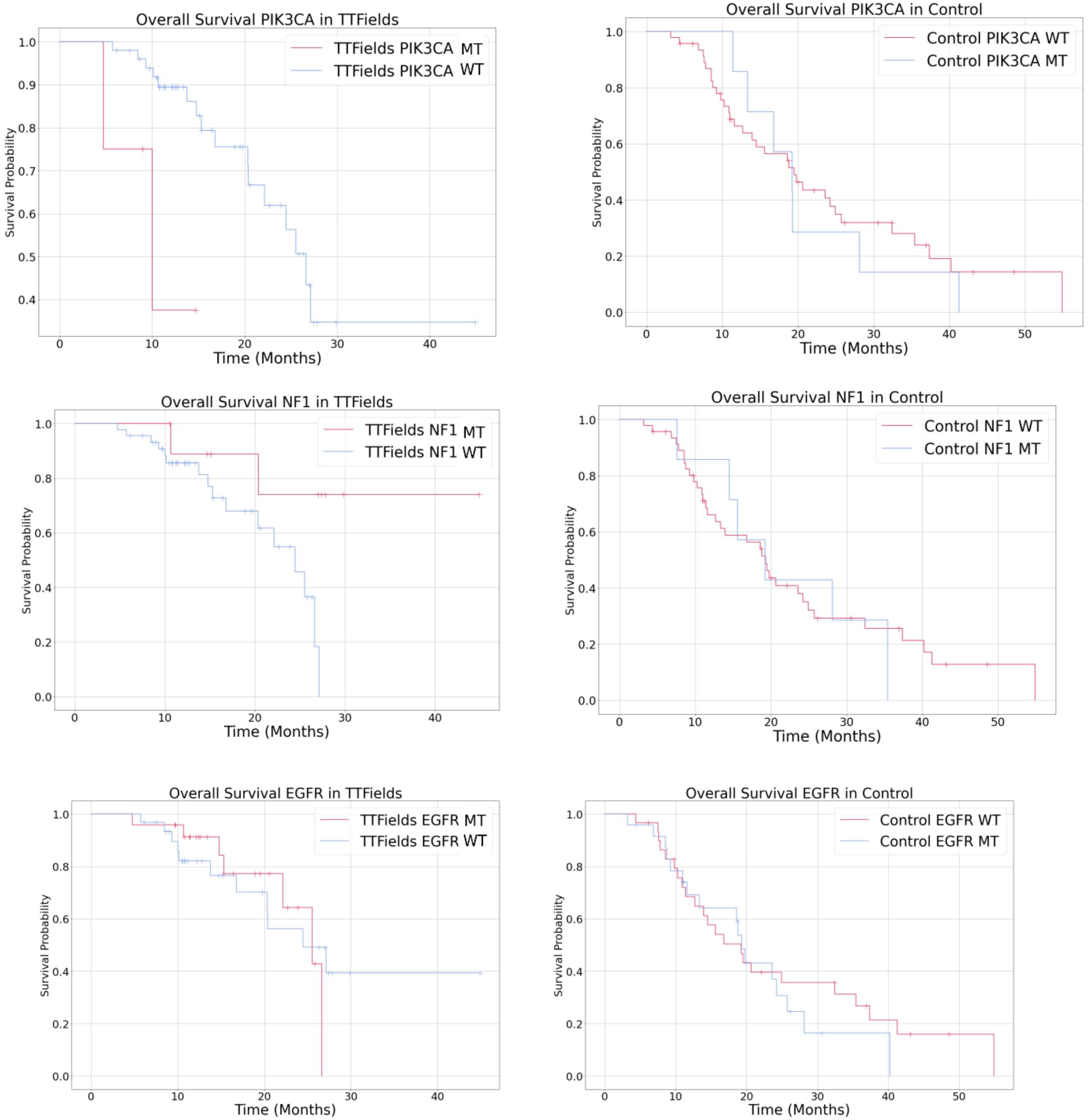

Supplemental Table 1

	Progression Free Survival		Interaction p-value (Biomarker and therapy)	Overall Survival		Interaction p-value (Biomarker and therapy)
	TTFields treated	Control		TTFields treated	Control	
**PIK3CA Mutated**	16.8m	11.2m	0.0200	10.0m	19.2m	*0.1196*
**PIK3CA Wild type**	6.7m	6.0m		26.6 m	19.5m	
P values [Cox PH]	*0.0008*	*0.6541*		*0.0158*	*0.6695*	
HR	*7.33*	*0.83*		*7.38*	*1.20*	
Lower 95%	*2.29*	*0.36*		*1.46*	*0.53*	
Upper 95%	*23.53*	*1.88*		*37.40*	*2.72*	
**NF1 Alteration**	18.2m	6.9m	0.3549	NR	19.3m	0.1412
**NF1 Intact**	14.4m	6.9m		24.7m	19.2m	
P values [Cox PH]	*0.0744*	*0.8933*		*0.0415*	*0.8902*	
HR	*0.45*	*1.06*		*0.20*	*1.06*	
Lower 95%	*0.19*	*0.47*		*0.04*	*0.44*	
Upper 95%	*1.08*	*2.40*		*0.94*	*2.56*	
**EGFR Mutated**	12.6m	6.9m	0.7821	25.3m	19.2m	0.5290
**EGFR Intact**	17.2m	6.9m		24.5m	19.3m	
P values [Cox PH]	*0.3628*	*0.3776*		*0.9462*	*0.5149*	
HR	*1.37*	*1.32*		*0.97*	*1.24*	
Lower 95%	*0.70*	*0.71*		*0.36*	*0.65*	
Upper 95%	*2.69*	*2.49*		*2.58*	*2.39*	

Supplemental Figure 4

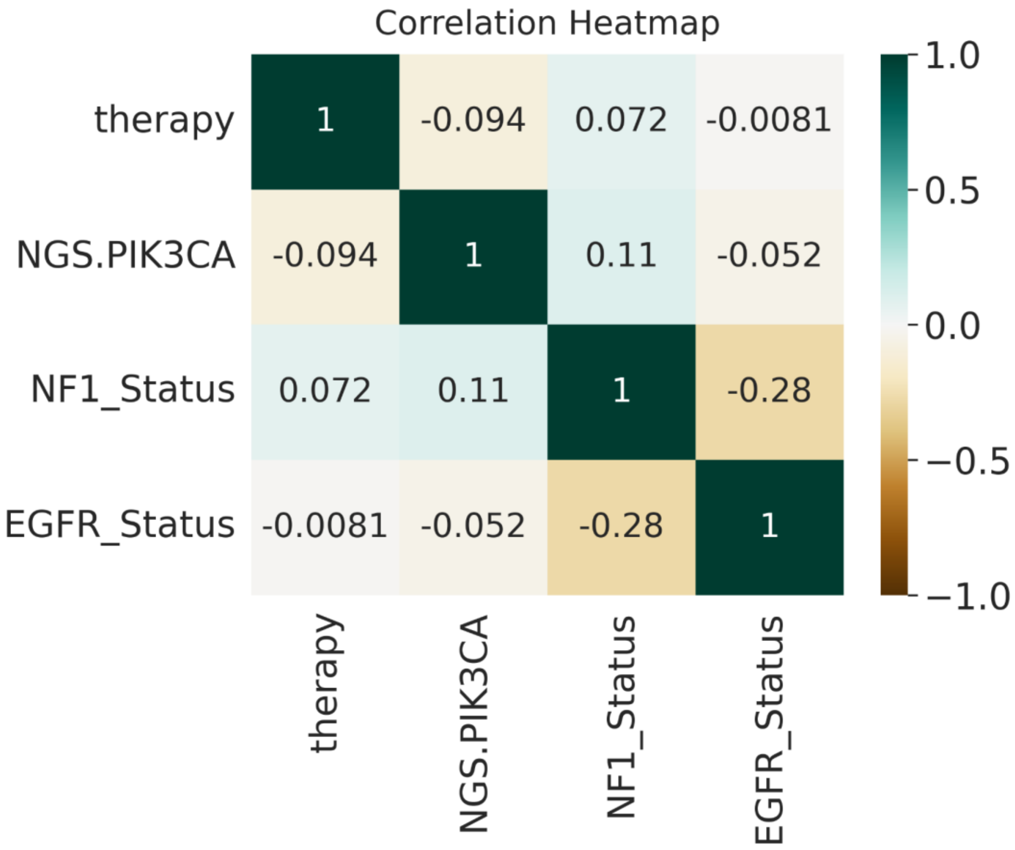

Supplemental Figure 5

	Progression Free Survival		Overall Survival	
Model	Concordance Index	Log Likelihood	Concordance Index	Log Likelihood
therapy + NGS.PIK3CA	0.62901701	-289.9317	0.59395349	-215.6307
therapy + NGS.PIK3CA + interaction	0.65193762	-287.60415	0.60914729	-214.64447
therapy + NF1_Status	0.63114367	-289.7176	0.59968992	-215.40537
therapy + NF1_Status + interaction	0.63397921	-289.29269	0.59023256	-214.18374
therapy + EGFR_Status	0.63031664	-289.26386	0.5772093	-216.05442
therapy + EGFR_Status + interaction	0.63031664	-289.22558	0.59364341	-215.85441
therapy + NGS.PIK3CA + NF1_Status	0.63575142	-289.45943	0.61736434	-214.61374
therapy + NGS.PIK3CA + EGFR_Status	0.63480624	-289.10403	0.5924031	-215.52514
therapy + NF1_Status + EGFR_Status	0.63610586	-289.09718	0.58728682	-215.39743
therapy + NGS.PIK3CA + NF1_Status + EGFR_Status	0.64189509	-288.86793	0.60186047	-214.60448

PFS model: therapy + NGS.PIK3CA + NF1_Status + EGFR_Status

covariate	HR	Lower 95%	Upper 95%	p
EGFR_Status	1.29955674	0.81044579	2.08385034	0.27677375
NF1_Status	0.80728039	0.43412151	1.50119632	0.49879188
NGS.PIK3CA	1.27013305	0.6457239	2.49834018	0.48844692
therapy	0.61683048	0.38110215	0.9983671	0.04922752

Supplemental Figure 6

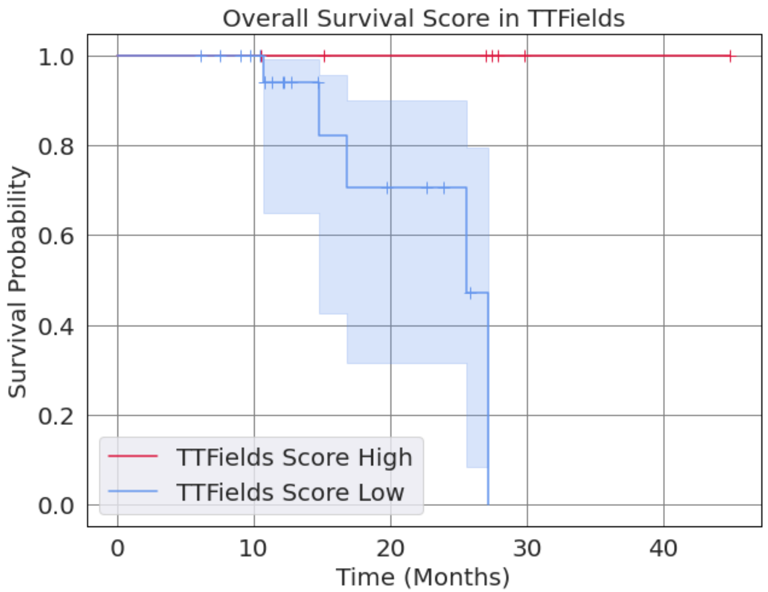

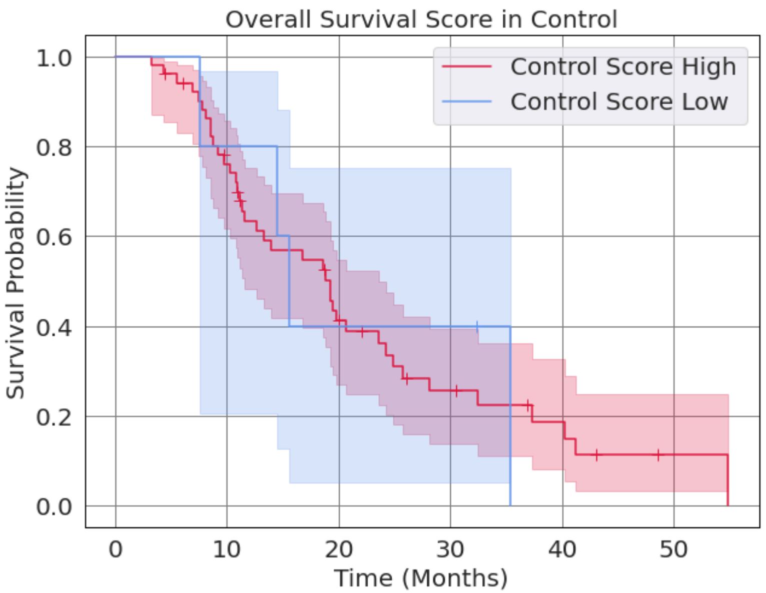

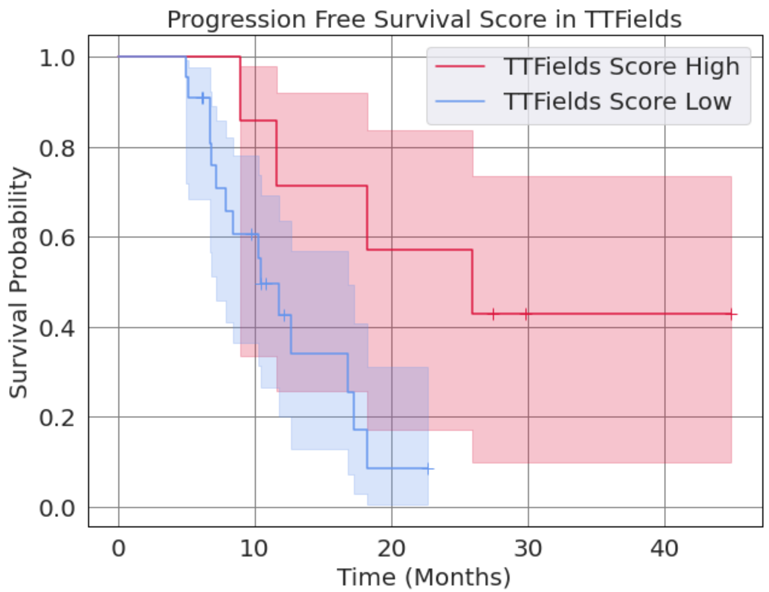

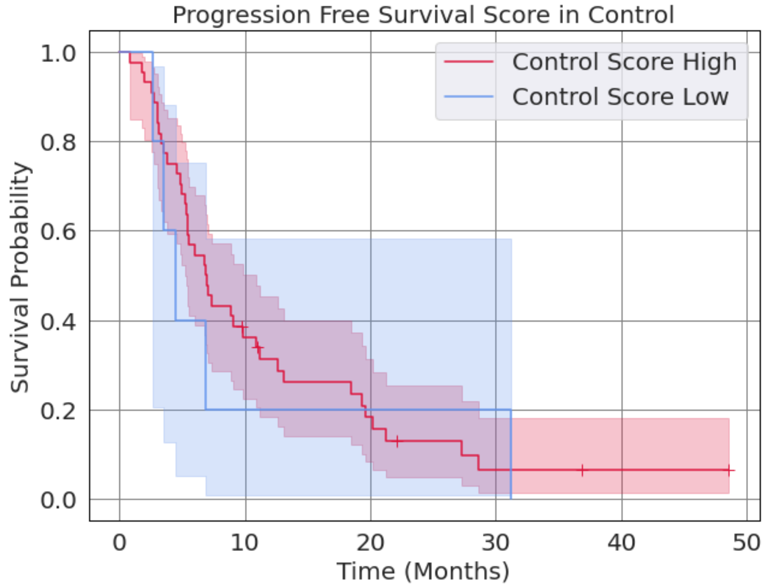

	> 50% compliance group					
	Progression Free Survival			Overall Survival		
	TTFields treated	Control	Interaction p-value (Score and therapy) 0.1173	TTFields treated	Control	Interaction p-value (Score and Therapy) 0.9950
**Score High**	25.9m	7.0m		NR	19.2m	
**Score Low**	10.4m	4.5m		25.5	15.6m	
P values [Cox PH]	0.0340	0.6485		0.9960	0.9847	
**HR**	0.25	1.25		1.01E-08	0.99	
**Lower 95%**	0.07	0.48		0	0.35	
**Upper 95%**	0.90	3.22		inf	2.79	

Supplemental Figure 7

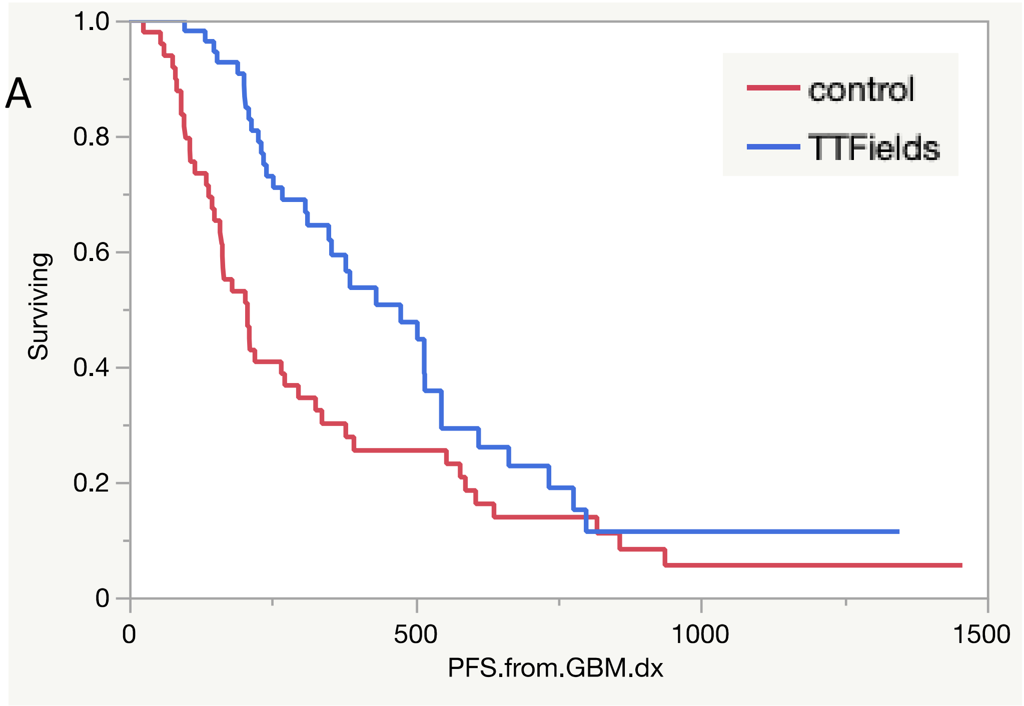

HR: 0.549, [95%CI: 0.35-0.86], p=0.0087

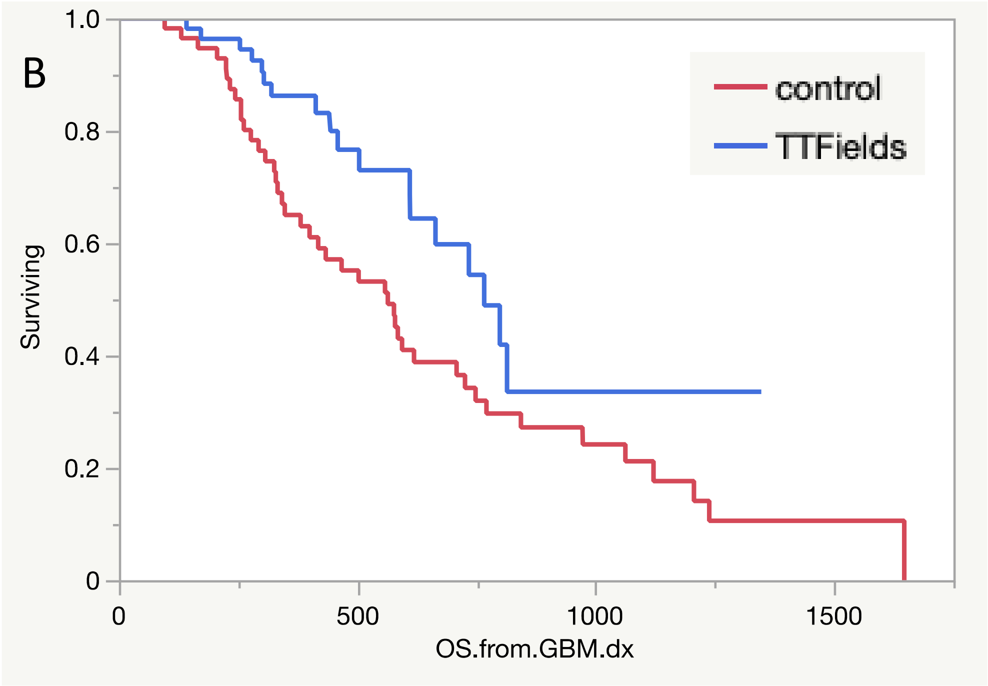

HR: 0.54 [95%CI: 0.31-0.95], p=0.0304

Supplemental Figure 7

C

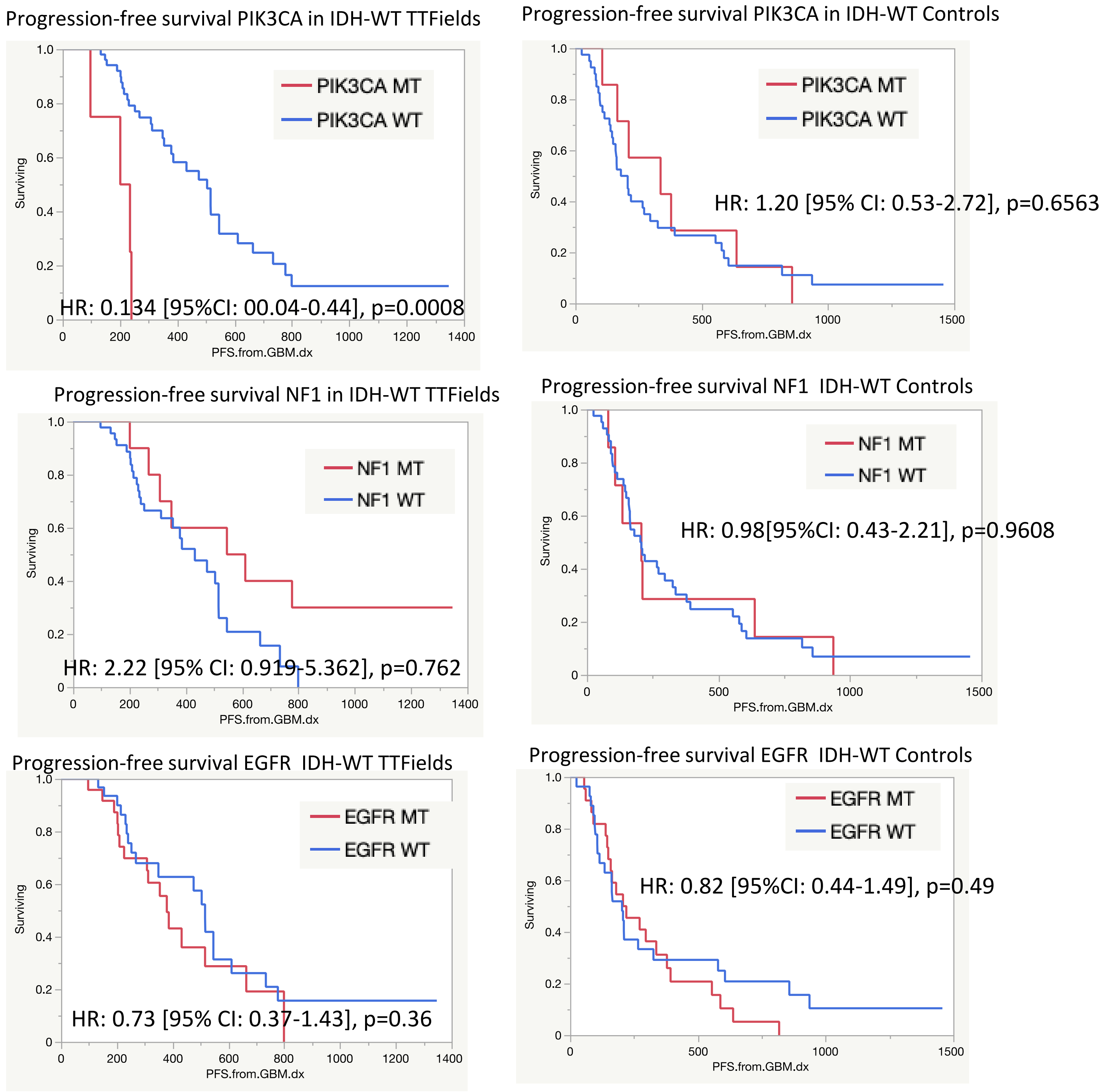

Supplemental Figure 7

D

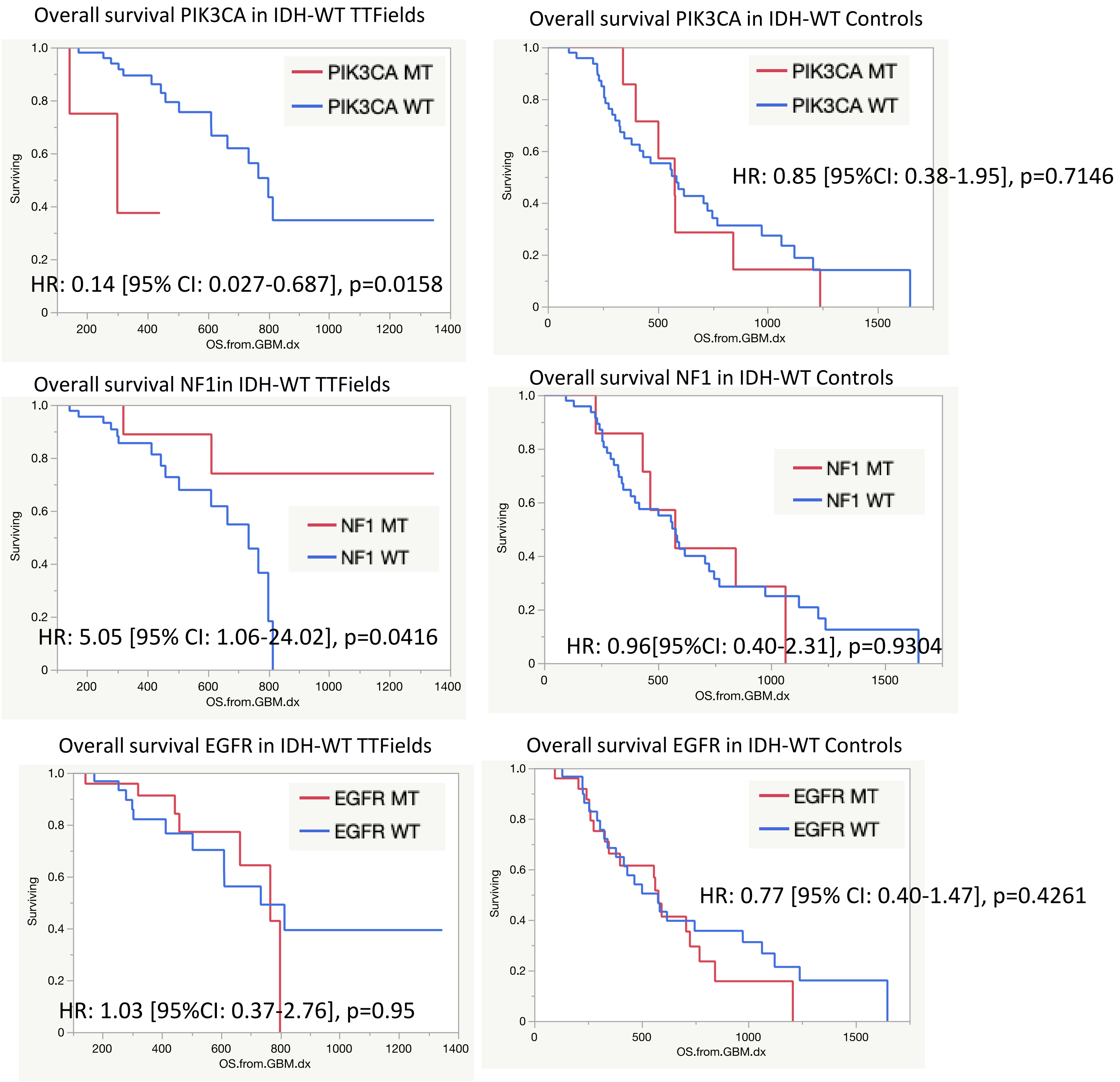

Supplemental Figure 7

E

Overall survival Score high vs. low in IDH-WT TTFields

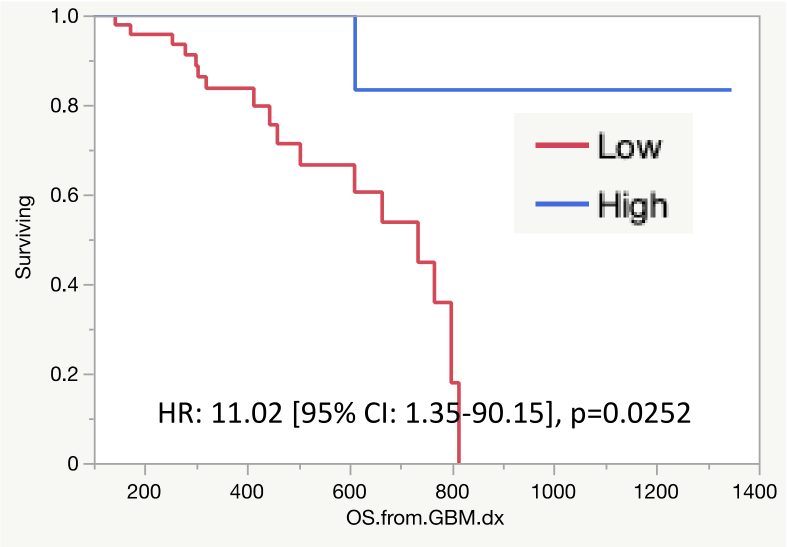

Overall survival Score high vs. low in IDH-WT Controls

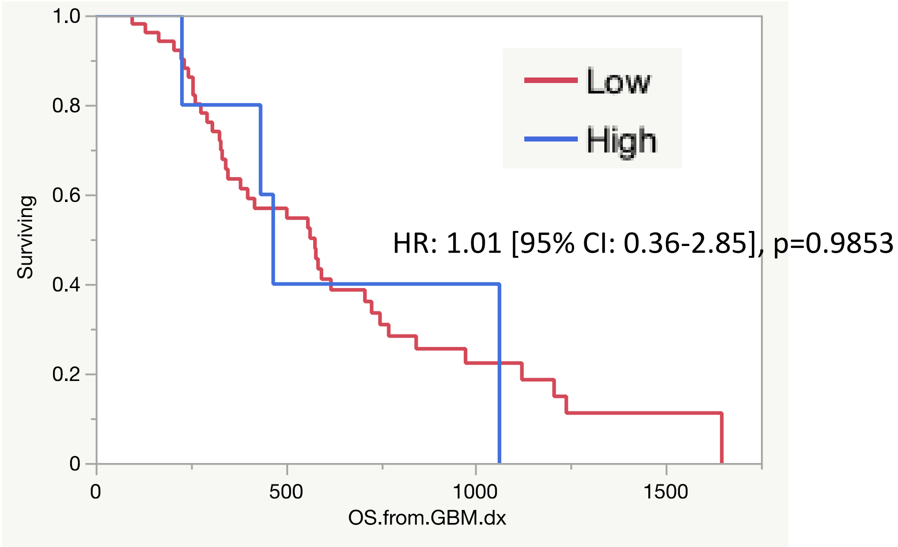

Progression-free survival Score high vs. low in IDH-WT TTFields

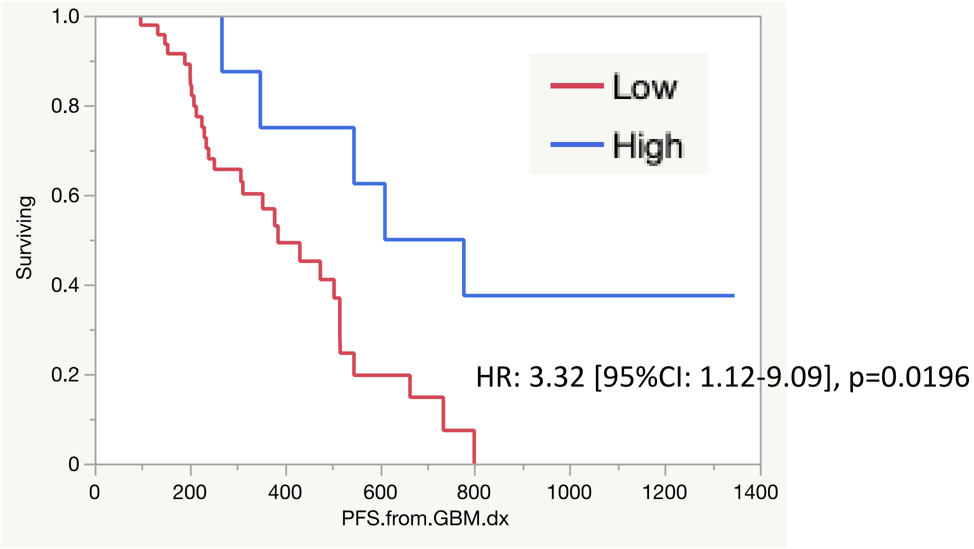

Progression-free survival Score high vs. low in IDH-WT Controls

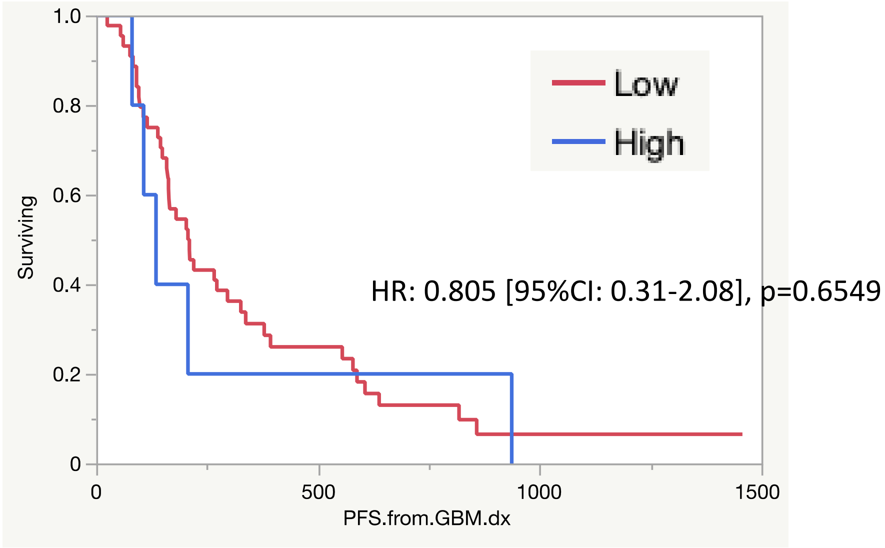

Supplement: vdac096_suppl_Supplementary_Material [file vdac096_suppl_supplementary_material.pdf]
