# Supplementary material for: Molecular alterations associated with improved outcome in patients with glioblastoma treated with Tumor-Treating Fields
Source: Neurooncol Adv. 2022 Jun 21;4(1):vdac096. doi: 10.1093/noajnl/vdac096 (PMC9270729; doi:10.1093/noajnl/vdac096)
Supplement: vdac096_suppl_Supplementary_Table_S1 [file vdac096_suppl_supplementary_table_s1.pdf]

Supplemental table 1

	Progression Free Survival		Interaction p-value (Biomarker and therapy)	Overall Survival		Interaction p-value (Biomarker and therapy)
	TTFields treated	Control		TTFields treated	Control	
**PIK3CA Mutated**	6.7m	11.2m	0.0200	10.0m	19.2m	*0.1196*
**PIK3CA Wild type**	16.8m	6.0m		26.6 m	19.5m	
P values [Cox PH]	*0.0008*	*0.6541*		*0.0158*	*0.6695*	
HR	*7.33*	*0.83*		*7.38*	*1.20*	
Lower 95%	*2.29*	*0.36*		*1.46*	*0.53*	
Upper 95%	*23.53*	*1.88*		*37.40*	*2.72*	
**NF1 Alteration**	18.2m	6.9m	0.3549	NR	19.3m	0.1412
**NF1 Intact**	14.4m	6.9m		24.7m	19.2m	
P values [Cox PH]	*0.0744*	*0.8933*		*0.0415*	*0.8902*	
HR	*0.45*	*1.06*		*0.20*	*1.06*	
Lower 95%	*0.19*	*0.47*		*0.04*	*0.44*	
Upper 95%	*1.08*	*2.40*		*0.94*	*2.56*	
**EGFR Mutated**	12.6m	6.9m	0.7821	25.3m	19.2m	0.5290
**EGFR Intact**	17.2m	6.9m		24.5m	19.3m	
P values [Cox PH]	*0.3628*	*0.3776*		*0.9462*	*0.5149*	
HR	*1.37*	*1.32*		*0.97*	*1.24*	
Lower 95%	*0.70*	*0.71*		*0.36*	*0.65*	
Upper 95%	*2.69*	*2.49*		*2.58*	*2.39*	
