# Supplementary material for: Molecular alterations associated with improved outcome in patients with glioblastoma treated with Tumor-Treating Fields
Source: Neurooncol Adv. 2022 Jun 21;4(1):vdac096. doi: 10.1093/noajnl/vdac096 (PMC9270729; doi:10.1093/noajnl/vdac096)
Supplement: vdac096_suppl_Supplementary_Legends [file vdac096_suppl_supplementary_legends.docx]

Supplemental Figure 1: Progression-free and Overall survival rates stratified by compliance (cutoff=0.50) to use of the TTFields (Optune) device.

Supplemental Figure 2: Progression-free and Overall survival rates stratified by compliance (cutoff=0.75) to use of the TTFields (Optune) device.

Supplemental Figure 3: PFS [A] and OS [B] of patients treated with TTFields vs control stratified for alterations of PIK3CA, NF1, and EGFR alterations.

Supplemental Table 1. Correlation and statistical comparisons of patients treated with TTFields vs control and listed by alterations of PIK3CA, NF1, and EGFR alterations.

Supplemental Figure 4. Correlation of PIK3CA, NF1, and EGFR with TTFields therapy.

Supplemental Figure 5: Model selection and the best model for the four variables

Supplemental Figure 6: Stratification of PFS and OS based on a combined Molecular Survival Score (MSS) for comparison of PFS and OS between TTFields vs control groups, for device compliance/usage rate >50%. The OS, PFS and statistical comparisons in all patients are shown here.

Supplemental Figure 7. Subgroup analysis in IDH WT tumors. [A, B]: PFS and OS of TTFields-treated and control patients who are IDH-Wild Type. [C, D]: PFS and OS in IDH-WT patients treated with TTFields vs control stratified for alterations of PIK3CA, NF1, and EGFR alterations. [E], Stratification of PFS and OS based on a combined Molecular Survival Score (MSS) calculated from the status of PIK3CA, NF1, and EGFR for comparison of PFS and OS between IDH-WT patients treated with TTFields vs control groups.
